# Changes in proteome and protein carbonylation in potato (*Solanum tuberosum* L.) under single and combined abiotic stresses

**DOI:** 10.1038/s41598-025-21439-y

**Published:** 2025-10-28

**Authors:** Marta Gietler, Justyna Fidler, Jakub Graska, Dominika Boguszewka-Mańkowska, Małgorzata Nykiel

**Affiliations:** 1 Department of Biochemistry and Microbiology, Institute of Biology, Warsaw University of Life Sciences-SGGW, Building 37, Nowoursynowska St. 159, Warsaw, 02-776 Poland; 2 Plant Breeding and Acclimatization Institute - National Research Institute in Radzików, Jadwisin Division, Department of Potato Agronomy, Szaniawskiego Str. 15, Serock, 05-140 Poland

**Keywords:** Abiotic stress, Carbonylation, Chloroplast, Potato, Proteasome 20S, Proteome, Biochemistry, Plant sciences

## Abstract

Environmental stresses are one of the main factors limiting plant production. In the era of a changing climate, more crop areas are exposed to drought and heat. The potato is one of the key plants for ensuring food security in the world, but at the same time, it is susceptible to environmental stresses. Our research aimed to investigate the response of potato to drought, increased temperature, and a combination of these stresses at the proteomic level. We also examined physiological parameters such as relative water content, assimilation surface and yield, as well as biochemical parameters such as the content of carbonylated proteins or 20 S proteasome activity. Our results demonstrated that abiotic stressors significantly affected photosynthesis and carbohydrate metabolism in potato plants. All tested treatments activated a series of proteins involved in the stress response, many of which were associated with chloroplasts. The responses to drought, high temperature, and their combination were distinct. Among these factors, high temperature exhibited the least detrimental effect on potato metabolism, which correlated with the absence of significant yield loss. Furthermore, the response to the combined stress closely mirrored the response to drought alone, suggesting that drought was the primary stressor influencing metabolic changes.

**Supplementary Information:**

The online version contains supplementary material available at 10.1038/s41598-025-21439-y.

## Introduction

The potato (*Solanum tuberosum* L.) is the third most consumed food crop worldwide, following rice and wheat. The FAO recognized the potato as “food for the future,” emphasizing its vital role in addressing future challenges related to global food security, nutrition, and poverty alleviation^1^. It is a highly nutritious crop, providing carbohydrates, minerals, vitamins, proteins, and high-quality dietary fiber. Due to its essential role in food and nutrition security, potatoes are a primary food source for many populations in various European and Latin American countries^1^. The sensitivity of potatoes to drought stress results from their shallow root system, which limits their ability to effectively absorb water from deeper soil layers^2^. Critical water demand occurs especially during the tuber growth phase, where water deficiency can lead to stunted growth, reduced yield quality, and decreased crop productivity, which is crucial from an economic point of view^3^. Drought often co-occurs with high temperatures, resulting in a synergistic interaction between both abiotic factors. The simultaneous occurrence of these stresses amplifies their negative effects, influencing fundamental physiological processes in the plant, such as gas exchange and photosynthesis. Drought causes a decrease in the water potential of cells, leading to stomatal closure, which reduces transpiration and limits gas exchange^4^. High temperature, on the other hand, increases water loss through enhanced transpiration, further disrupting the plant’s water balance. Both factors also affect cellular respiration, leading to disturbances in the plant’s energy metabolism^5^. Effects of singular stresses: drought and heat on potato plants were reported several times in many aspects: from morphological, physiological, to molecular studies^6,7^, but their combination is rarely studied. It has been proven that abiotic stresses, such as drought and high temperature, significantly affect metabolic processes, including glycolysis, a key pathway responsible for ATP production^8^. Under drought, the activity of glycolytic enzymes changes, affecting carbohydrate metabolism and causing energy distribution in the plant. Increased accumulation of glycolytic products, such as phosphoenolpyruvate and pyruvate, may support the biosynthesis of osmolytes, such as soluble sugars^9^. Plants also accumulate other osmolytes, such as proline, which help maintain osmotic balance and protect cell structures from damage^10^. Water deficiency leads to stomatal closure, limiting carbon dioxide access and thereby inhibiting photosynthesis^11^. The activity of photosynthetic enzymes is reduced, causing disturbances in the Calvin cycle, which results in limited production of ATP and NADPH, contributing to inhibited plant growth^12^.

Moreover, in response to abiotic stresses, plants activate a network of defense mechanisms, consisting of specific proteins and enzymes, among others. In high-temperature stress, heat shock proteins (HSPs) play a crucial role by stabilizing the structure of other proteins, preventing their denaturation^13^. The plant’s response to stress also includes the activation of transcription factors, such as DREB (dehydration-responsive element-binding proteins) and HSF (heat shock factors), which initiate the expression of genes encoding protective proteins and repair enzymes^14^. Water stress, in particular, increases the activity of antioxidant enzymes, such as superoxide dismutase (SOD), catalase (CAT), and ascorbate peroxidase (APX), which neutralize reactive oxygen species (ROS)^15^.

Despite the activation of mechanisms aimed at reducing oxidative stress, the ROS content usually increases under drought and high temperature stresses, causing damage to lipids, nucleic acids, and proteins. One of the most occurring oxidative events is protein carbonylation — a process that destabilizes the structure and function of proteins. Plants reduce the effects of this adverse modification by activating proteolytic pathways, such as the 20 S proteasome, responsible for the selective degradation of carbonylated proteins^16^. This mechanism enables the regulation of oxidized protein levels, which is necessary for adaptation to stress.

Although metabolic responses of potato to individual stresses are relatively well understood, in natural environments drought or high temperature rarely occur in isolation. The co-occurrence of these two stresses is therefore a much more common problem, intensifying especially in the era of climate change, which causes an increase in the Earth’s average surface temperature and low precipitation in some areas^17^. Because the plant’s response to single and multi-stress conditions is different and is not the sum of the response to a single stress, existing studies on single stresses do not provide an answer to the response to double stress^18^. Currently, there are no studies demonstrating changes in the proteome of potato leaves exposed to the double stress of drought and high temperature. Comparison of changes in the proteome of potatoes exposed to drought or heat and to a combination of these stresses has so far only been described in studies on the root proteome^19^, and this will differ from the response in leaves.

What is more, changes in the abundance of individual proteins alone do not provide a complete picture of the likely changes in potato metabolism. This is due to the fact that, after biosynthesis, proteins undergo post-translational modifications that can modulate their properties^20^. Under stress conditions, protein carbonylation can occur, a modification that is considered a marker of oxidative stress^21^. As was mentioned above, carbonylated proteins generally lose their biological properties. As a result of carbonylation, they can be targeted for degradation or accumulated in the cell, but the formation of such aggregates is toxic^22^. Therefore, identifying proteins particularly susceptible to carbonylation is crucial. In this regard, the present study is also unique because it shows not only changes in the overall leaf proteome but also in the content of carbonylated proteins and indicates proteins that are targeted by this modification or, conversely, protected from it under the studied stress conditions.

In the face of ongoing climate change, which increases the frequency and severity of extreme weather events, understanding plant responses to abiotic stresses is crucial. Research on protective proteins, oxidative damage repair mechanisms, and proteasome activity lays the foundation for developing new breeding strategies. Molecular selection and agricultural biotechnology can contribute to creating potato varieties with enhanced resistance to environmental stresses, which is essential for ensuring stable yields under changing climatic conditions^23^.

The aim of this study was to analyze the responses of potato to abiotic stressors - drought and high temperature and their combination. Two-dimensional gel electrophoresis (2DE) was used to analyze changes in protein profiles and carbonyloproteome, and the degree of protein damage was assessed by measuring total protein carbonylation. Moreover, the degradation of carbonylated proteins was evaluated by measuring proteasome 20 S activity. This study contributes to a better understanding of plant defense mechanisms to not only singular stresses of drought and heat but also to double stress, which is far more common in a natural environment.

## Results

**Fig. 1:**
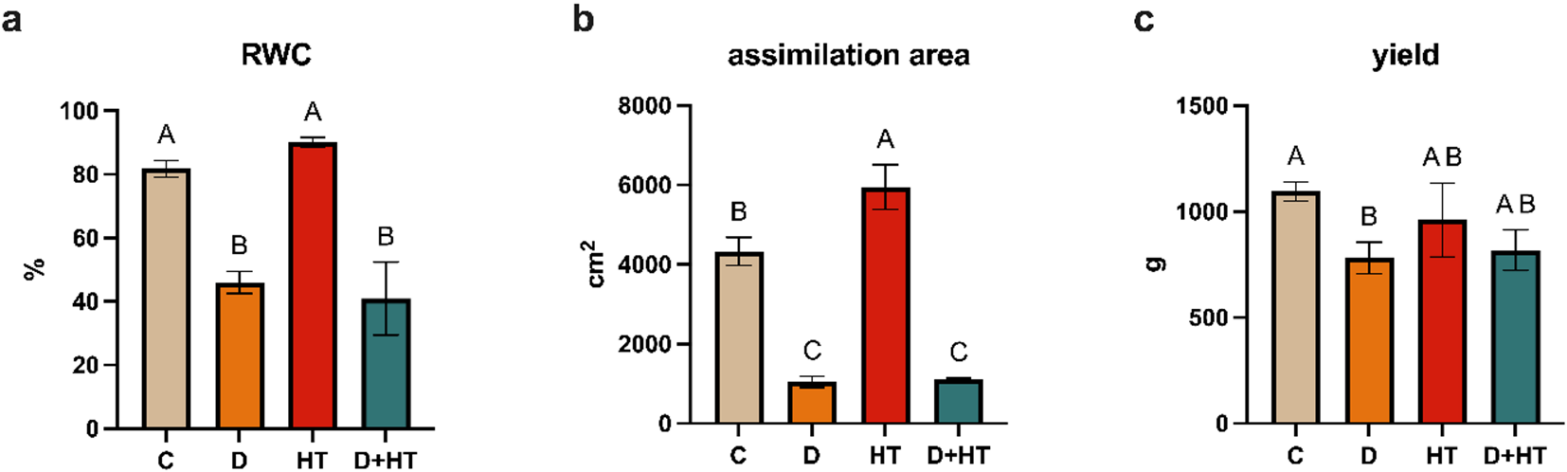
Plant parameters: RWC (**a**), assimilation area per plant (**b**), yield per plant (**c**). C- control; D- drought; HT- high temperature. Results are shown as the means ± SD. Different letters indicate homogeneous groups that are significantly different at *p* < 0.05 according to a two-way analysis of variance and a post-hoc Tukey’s test.

The water content in the aboveground tissue changed under the influence of stresses (Fig. 1a). As a result of high temperature, an increase in relative water content (RWC) by 8% was observed. On the other hand, drought caused a significant decrease in RWC to 46%. The double stress of drought and high temperature had the strongest effect on RWC, causing a reduction in the value of this parameter by over 50% compared to the control, reaching about 34%. Thus, a high temperature significantly deepened tissue dehydration in drought conditions. Similar changes were observed in the assimilation surface (Fig. 1b). Under the influence of high temperature, the assimilation surface increased by 37%, while both drought stress and double stress caused a decrease in the assimilation surface by about 75%. These changes were reflected in the obtained yield (Fig. 1c). While high temperature did not significantly affect the obtained potato yield, both drought and double stress caused a yield reduction by 28% and 25%, respectively.

**Fig. 2:**
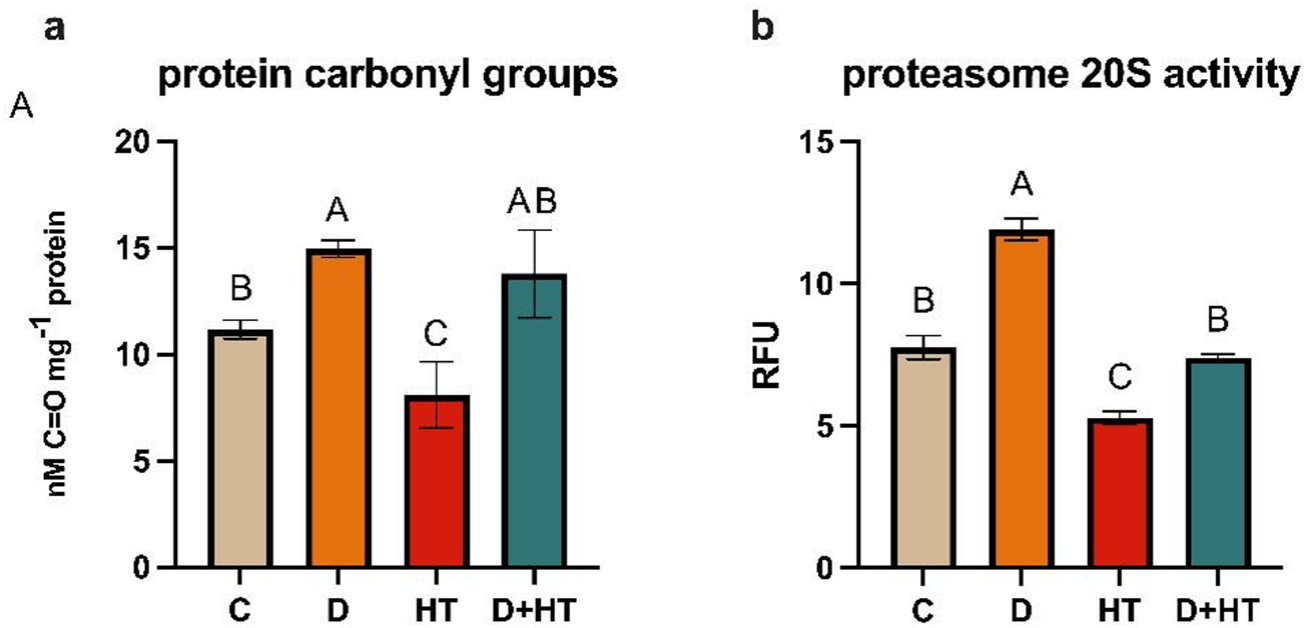
Protein carbonylation (**a**) and proteasome 20 S activity (**b**). C- control; D- drought; HT- high temperature. Results are shown as the means ± SD. Different letters indicate homogeneous groups that are significantly different at *p* < 0.05 according to a two-way analysis of variance and a post-hoc Tukey’s test.

Stress significantly affected the content of carbonylated proteins (Fig. 2a). A decrease in the content of oxidized proteins was observed only as a result of high temperature and amounted to about 30%. Both drought and high temperature increased the accumulation of carbonylated proteins, with an increase of 34% on average in drought and 23% in double stress compared to the control. One of the most important ways of removing carbonylated proteins is their degradation in ubiquitination independent manner by the 20 S proteasome. At high temperature, the activity of the 20 S proteasome decreased in comparison to the control by 32%, and in drought increased by 53%. High protein carbonylation in drought and their simultaneous rapid removal by the 20 S proteasome may indicate intensive oxidation of cell components in this stress. In the double stress of drought and high temperature, the activity of the 20 S proteasome was similar to the control (Fig. 2b).

**Fig. 3:**
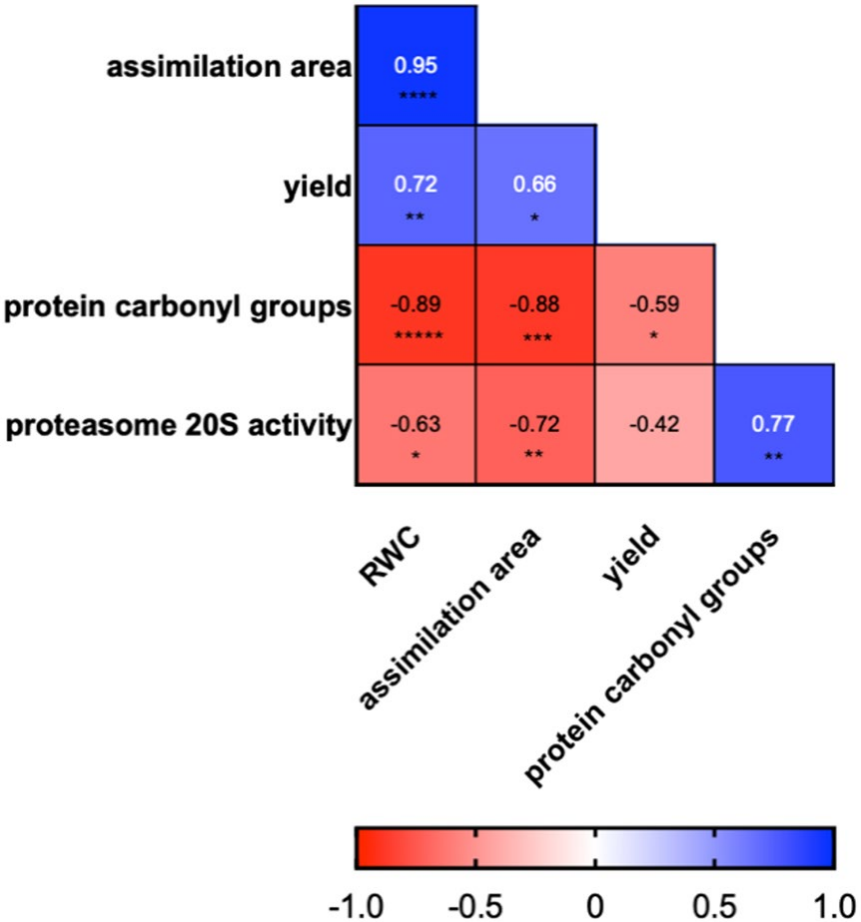
A heat map showing correlation coefficients between RWC, assimilation area, yield, proteasome 20 S activity, and protein carbonyl groups. Blank space means value of correlation close to zero. ****—a significance level of *p* ≤ 0.0001, ***—a significance level of *p* ≤ 0.001, **—a significance level of *p* ≤ 0.01, *—a significance level of *p* ≤ 0.05.

Numerous interactions between the given parameters were demonstrated (Fig. 3). A very strong (*p* ≤ 0.0001) positive correlation coefficient was shown for RWC and the assimilation area (0.95), a strong positive correlation (*p* ≤ 0.01) was also found between the content of carbonylated proteins and 20 S proteasome activity (0.77), RWC and yield (0.72), and a significant correlation (*p* ≤ 0.05) between yield and assimilation area (0.66). The strongest negative correlation (*p* ≤ 0.0001) was shown for RWC and the content of carbonylated proteins (-0.89). A strong correlation (*p* ≤ 0.001) was found between assimilation area and the content of carbonyl groups in proteins (-0.88). A lower but significant negative correlation was also noted for 20 S protesome activity and assimilation surface (-0.72; *p* ≤ 0.01), 20 S protesome activity and RWC (-0.63; *p* ≤ 0.05), and yield and carbonylated protein content (-0.59; *p* ≤ 0.05).

Image analysis of total proteome maps by Delat2D revealed 547 protein spots on the master gel in the pH range 4–7 and the size range between 10 and 250 kDa (Suppl. 1).

Principal component analysis (PCA) of the proteomic maps revealed four distinct sets among the four replicates (Suppl. 2a). The first three components explained 49.404% of the variance, with notable differences in PC1 and PC3 between control (red) and high temperature (yellow) treatments, as well as between drought (blue) and double stress (green). In contrast, PCA for carbonylated proteins (Suppl. 2b) accounted for 42.767% of the variance, where only drought differed in PC1, while PC2 indicated separation of high temperature and drought from control and double stress.

For further analysis, only proteins with significant abundance change in comparison to the control were selected using one-way ANOVA with an adjusted Bonferroni correction (critical p-value < 0.05). Analysis showed 57 differently abundant proteins, which were then identified by LC-MS/MS. In drought alone, 24 of differently abundant proteins were down-regulated, and 33 were up-regulated, while in heat and double stress, 22 proteins were down-regulated and 35 were up-regulated.

**Fig. 4:**
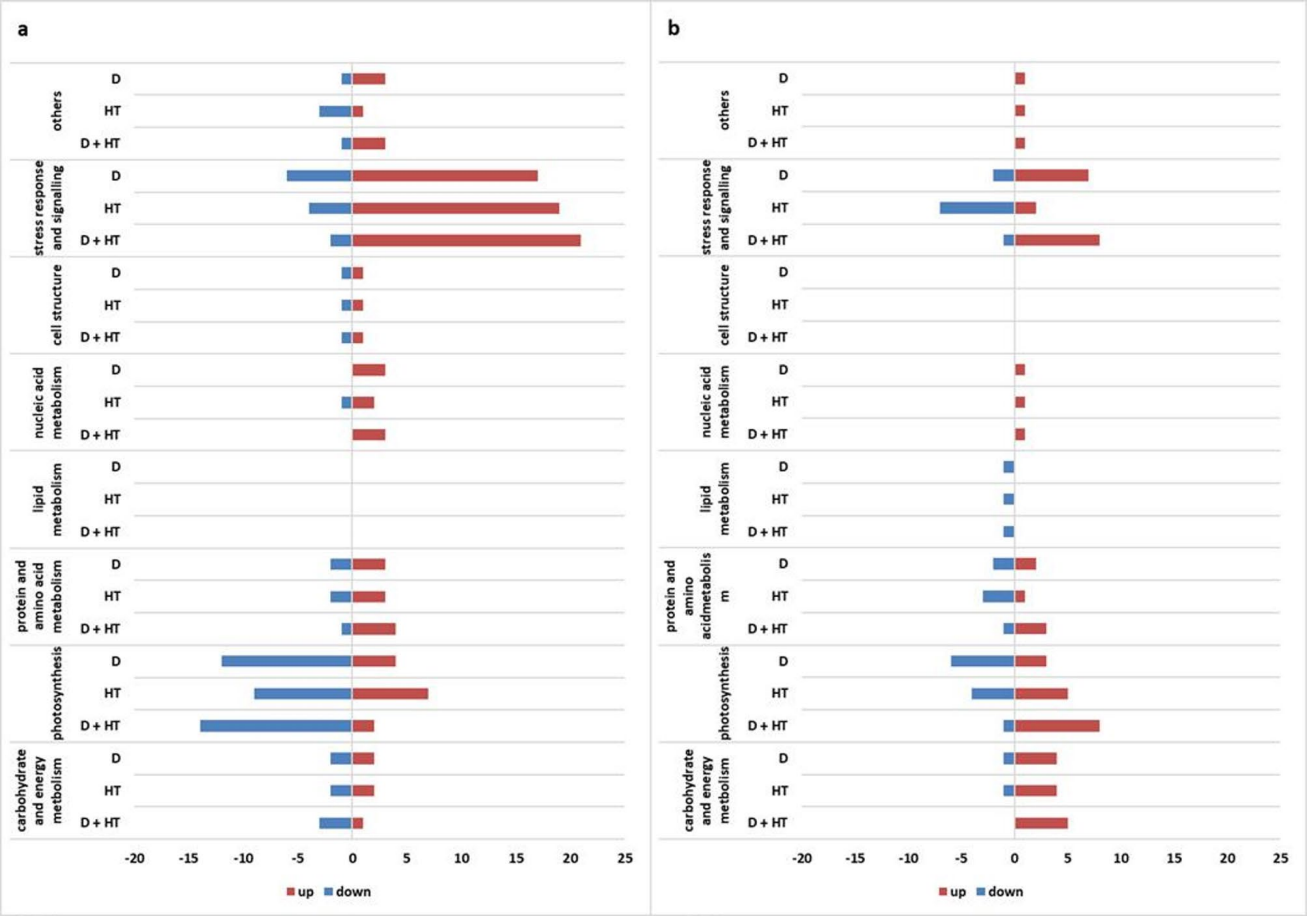
Groups of differentially abundant proteins: (**a**) differentially abundant protein in total proteome; (**b**) - differentially abundant carbonylated proteins. C- control; D- drought; HT- high temperature.

Differential proteins were categorized by metabolic processes (Table 1; Fig. 4.a), showing significant changes in proteins related to stress response and signaling, and a decline in photosynthesis proteins. Notably, double stress led to up-regulation of 21 out of 23 stress response proteins, while drought caused up-regulation of only 17. Photosynthesis proteins were most down-regulated under double stress (14 out of 16) and the least in high temperature (9 proteins).

Image analysis of total carbonylated proteins’ maps by Delat2D revealed 167 protein spots on the master blot in the pH range 4–7 and the size range between 10 and 250 kDa (Suppl. 3). For further analysis, only proteins with significant abundance change in comparison to the control were selected using one-way ANOVA with an adjusted Bonferroni correction (critical p-value < 0.05). Analysis showed 30 differently abundant carbonylated proteins, which were then identified by LC-MS/MS. In drought alone, 12 of the differently abundant carbonylated proteins were down-regulated, and 18 were up-regulated; in heat, 16 proteins were down-regulated and 14 were up-regulated; in double stress, 4 were down-regulated and 26 were up-regulated.

For carbonylated proteins (Table 2; Fig. 4b), stress response and photosynthesis predominated. Most stress response proteins were up-regulated in drought (7 out of 9) and double stress (8 out of 9), whereas heat saw reduced levels in 7 out of 9. The carbonylated proteins related to photosynthesis varied, with drought showing mostly down-regulation (6 out of 9), high temperature an increase (5 out of 9), and double stress the highest (8 out of 9). Similar trends were noted in carbohydrate metabolism, with increased carbonylation in double stress.

**Table 1:**
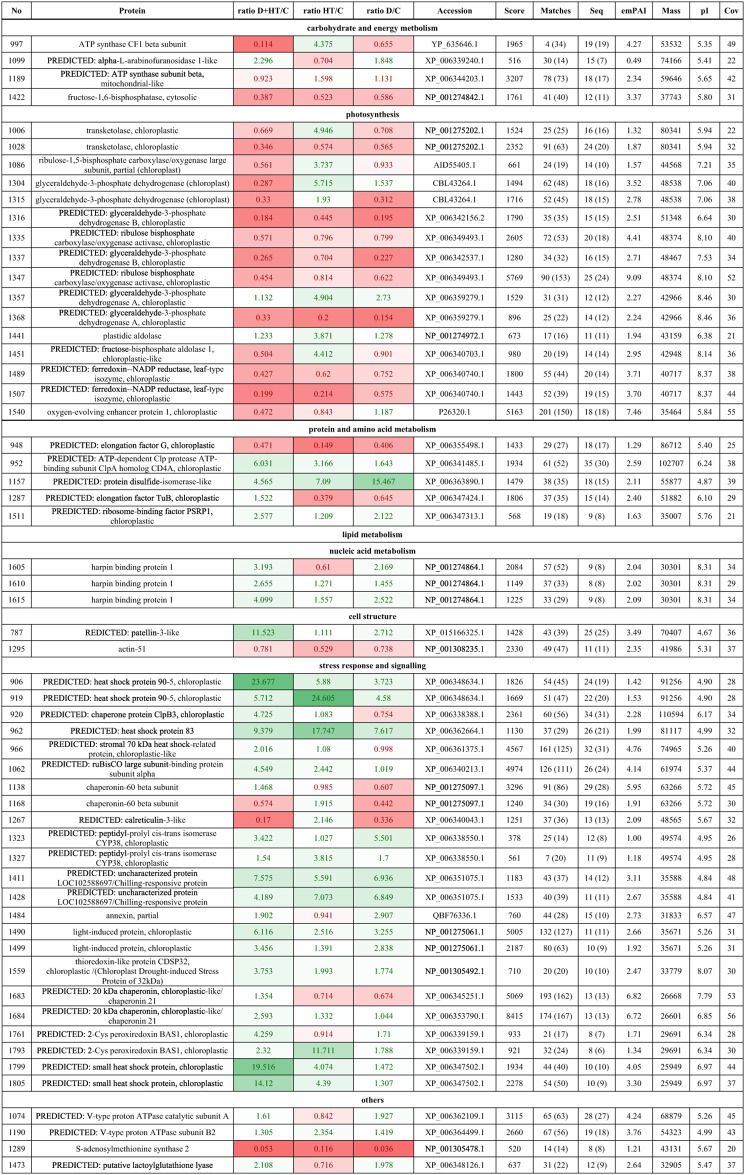
Differentially abundant proteins identified from selected spots by LC–MS/MS in Solanum tuberosum (L.). Abbreviations: No- number assigned to spot, Ratio- abundance of proteins in drought (D), high temperature (HT), and combination of those stresses (D+HT) to control (C) plants; Seq- sequences, emPAI-Exponentially modified protein abundance index, pI- isoelectric point, Cov- coverage. Fields marked in red: down-regulated proteins. Fields marked in green - up-regulated proteins. The intensity of the color indicates the level of the differences

**Table 2:**
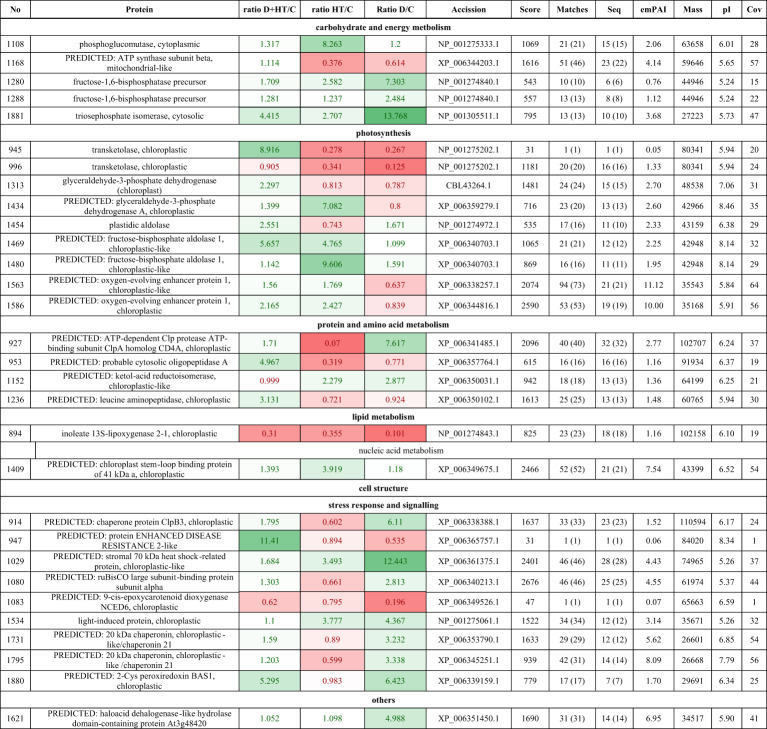
Differentially abundant carbonylated proteins identified from selected spots by LC–MS/MS in Solanum tuberosum (L.). Abbreviations: No- number assigned to spot, Ratio- abundance of proteins in drought (D), high temperature (HT), and combination of those stresses (D+HT) to control (C) plants; Seq- sequences, emPAI-Exponentially modified protein abundance index, pI- isoelectric point, Cov- coverage. Fields marked in red: down-regulated proteins. Fields marked in green - up-regulated proteins. The intensity of the color indicates the level of the differences

**Fig. 5:**
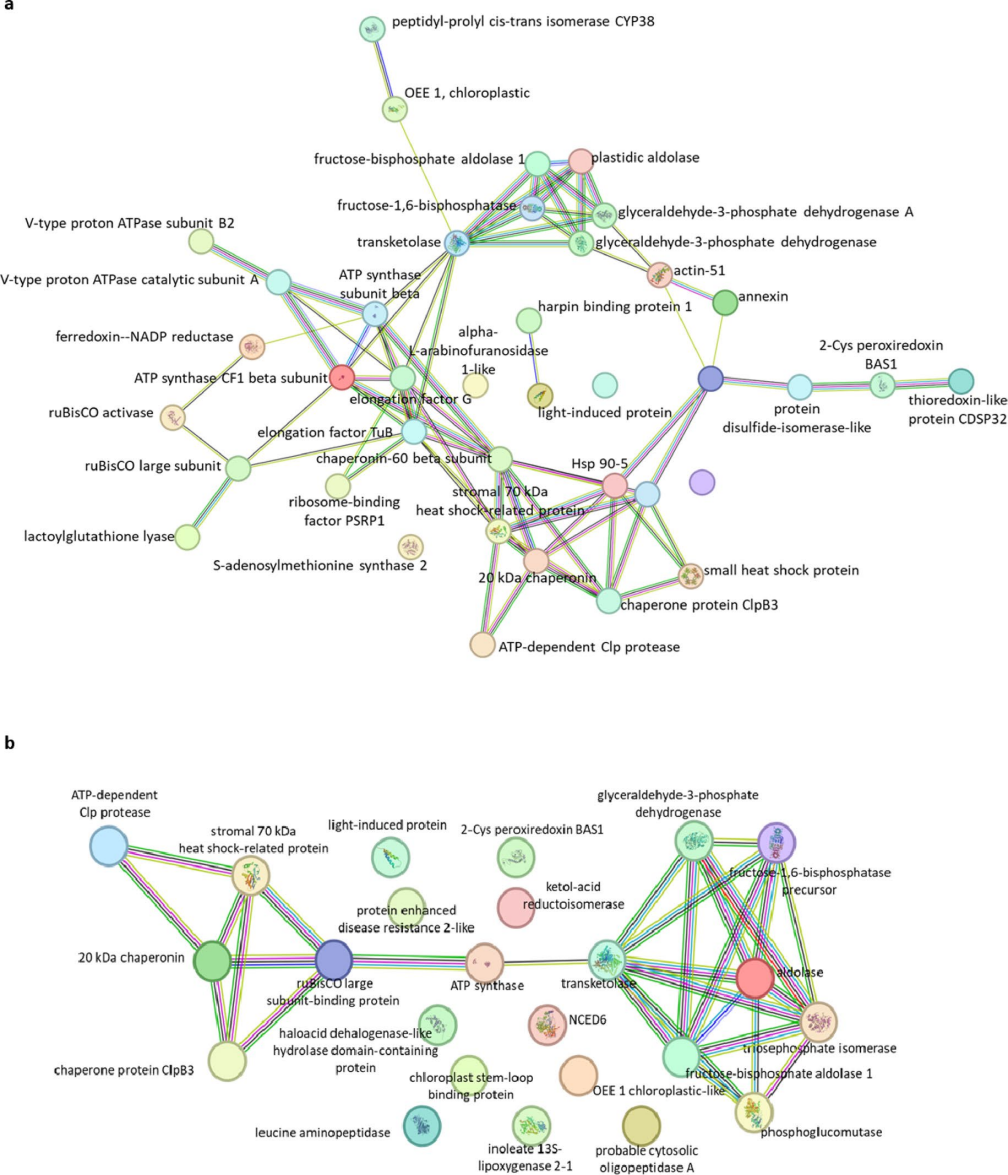
Proteins relationship networks of differently abundant proteins (**a**), and differently abundant carbonylated proteins (**b**) by STRING software version 12.0, accessible online (https://string-db.org), a database of known and predicted protein interactions (PPIs). Search performed for multiple proteins, with protein interaction level set at 0.400. Edges: known interactions: cyan - from curated databases, magenta - experimentally determined; predicted interactions: green - gene neighborhood, red - gene fusions, blue -gene co-occurrence; others: lime – textmining, black - co-expression, purple - protein homology.

STRING analysis of the connections between proteins showed that the identified differential proteins form an extensive network of interdependencies, which suggests a systemic response to the analyzed stresses (Fig. 5a). Protein clusters are visible, in particular a cluster of proteins related to carbohydrate metabolism, which is connected to another cluster of proteins related to photosynthesis and energy production. Another coherent group is formed by proteins with chaperone activity. Among the differentially abundant carbonylated proteins (Fig. 5b), only two clusters are visible: proteins related to sugar metabolism, and proteins with chaperone activity. The remaining identified differential proteins seem not to have a strong connection with each other.

## Discussion

Drought conditions often lead to reduced yields due to a decrease in assimilatory leaf surface area, inhibiting photosynthesis and causing stomatal closure to limit water loss^24^. Interestingly, potato plants did not experience decreased yields under elevated temperatures, possibly due to the maintenance of assimilatory surface area. While high temperatures can accelerate metabolism and transpiration, adequate water availability allows for retained photosynthetic capacity. However, combined stress from drought and heat resulted in decreased leaf area and yields, as the plant struggled to cope with dual stressors, exacerbating water deficits and lowering photosynthetic efficiency.

Abiotic stresses increase the production of reactive oxygen species, overwhelming the cell’s defenses^25,26^. This leads to oxidative damage, such as carbonylation of proteins, which impairs essential cellular functions due to the fact that carbonylated proteins usually lost their function^27^. Although recent studies indicate that carbonylation mediates in the transduction of ROS and phytohormone signals^21^. Increased content of carbonylated proteins was observed in potato plants under drought, while high temperatures reduced their level. The reduced protein carbonyl content under high-temperature stress may be due to improved protein protection against oxidative stress, which involved the activation of protein protective mechanisms, particularly HPSs and chaperonins^28^, which occurred under these conditions. Furthermore, reduced carbonylation of numerous chaperones was observed, which, combined with their higher content, may indicate a more efficient protein protection system against oxidation. A similar response was observed in wheat under drought conditions, where prolonged stress enabled the activation of defense mechanisms and a reduction in the content of carbonylated proteins^29^.

One of the specific pathways for carbonylated proteins removal is through proteasome 20 S. The activity of the 20 S proteasome varied with stress conditions and was linked to carbonylation levels. Under high temperatures, decreased carbonylation reduced the need for protein degradation. Similarly, an increase in 20 S proteasome activity was also associated with salinity stress as an adaptive mechanism, indicating its importance in coping with environmental stress^16^. Research has shown that certain genes related to the 20 S proteasome respond critically to abiotic stresses, highlighting their potential for breeding programs to obtain climate-resilient plants^30^.

One of the critical aspects of plant stress tolerance involves maintaining protein homeostasis and ensuring functional photosynthesis under adverse environmental conditions. Photosynthesis converts light energy into chemical energy, but abiotic stress factors negatively impact photosystem I (PSI) and photosystem II (PSII), electron transport chains, and chlorophyll production. These stresses lower stomatal conductance, leading to oxidative stress, which reduces ribulose-1,5-bisphosphate carboxylase/oxygenase (Rubisco) activity and hinders photosynthesis^31^. Drought stress notably decreases turgor pressure, causes stomatal closure, reduces gas exchange, CO_2_ assimilation, and impairs the photosynthetic apparatus while increasing metabolite flows^32^. Drought often coincides with high-temperature stress, further affecting photosynthetic enzyme activity, and excess light, which can cause photoinhibition, particularly damaging to PSII^33^.

Ferredoxin-NADP reductase (FNR) is crucial in the electron transport chain, transferring electrons from ferredoxin (Fd) to NADP^+^, facilitating NADPH formation, which is vital for CO_2_ fixation and antioxidant mechanisms^34^. However, under stress conditions, photoreduced Fd can divert electrons to the cytochrome b_6_f complex, initiating cyclic electron flow (CEF) that bypasses NADPH production, likely to meet ATP demands or relieve electron pressure during unfavorable conditions^35^. In potato leaves, stresses like heat and drought led to downregulation of chloroplastic FNR, similar findings were noted in tomato and tobacco^35,36^. This downregulation may trigger CEF, enhancing ATP synthesis crucial for plant function under stress, as ATP needs may outweigh demand for NADPH.

Oxygen-evolving enhancer protein 1 (OEE1), associated with PSII, stabilizes the manganese cluster critical for water-splitting reactions^37^. In our study, OEE1 showed over twofold reduction under combined stress and was carbonylated under high temperature, whereas it slightly increased under drought. Both reduction in abbundance and carbonylation act as a hindering factor. This suggests that OEE1 may have a protective role only under drought conditions. Also, CYP38, a peptidyl-prolyl *cis*-trans isomerase, increased in abundance with stress, indicating a protective function for PSII. Its absence in Arabidopsis resulted in impaired photochemical efficiency and increased reactive oxygen species production^38^. Additionally, mutations in CYP38’s ortholog disrupted chloroplast development and heightened sensitivity to light^39^.

The ATP synthase CF1 beta subunit in plant chloroplasts is crucial for ATP production during the light phase of photosynthesis. It converts proton gradient energy into ATP for the Calvin cycle^16^. Our research shows that its levels changed under abiotic stresses: high temperatures increased its abundance, indicating boosted ATP production, while drought and combined stresses decreased its levels, suggesting inhibited photosynthesis and energy conservation, however there is limited research on this enzyme’s role under stress conditions.

In double stress abundance of FNR, OEE1, and ATP synthase CF1 was significantly lower than in single stresses. What is more, in contrast to drought, carbonylation of OEE1 was increased. Therefore, the double stress of heat and drought may lead to enhanced ATP synthesis, but at the same time, impaired photochemical efficiency and increased ROS synthesis may occur.

One of the crucial defense mechanisms in plants involves the modification of key metabolic pathways, including the Calvin cycle, the pentose phosphate pathway (PPP), and glycolysis^40^. Environmental stresses often lead to the reprogramming of these pathways, enhancing resource utilization and maintaining homeostasis.

Rubisco is a key enzyme in the Calvin cycle, converting atmospheric CO_2_ into organic compounds. It consists of eight large (RbcL) and eight small (RbcS) subunits, with proper assembly critical for function. This assembly relies on chaperones that facilitate protein folding^41^. RbcL subunits are plastid-encoded, while RbcS subunits are nuclear-encoded. Cpn60 chaperonins assist RbcL folding and were initially thought to aid in assembly^42^. Chloroplast chaperonins are formed from Cpn60α and Cpn60β subunits in a 1:1. Our results showed upregulation of Cpn60α in all treatments, with a slight increase and intense carbonylation in drought-stressed plants. In double stress, Cpn60α content increased alongside carbonylation, while heat stress led to increased protein content but decreased carbonylation. This suggests compromised functionality of Cpn60α under drought and double stress. In tomato plants, drought stress resulted in reduced Cpn60α abundance and gene expression^43^. Kang et al.^44^ also reported downregulation in wheat, though higher abundance was noted upon salicylic acid pretreatment, indicating enhanced Rubisco activation and photosynthesis. In rice, *OsCpn60α1* and *OsCpn60α2* transcription increased with high temperatures, although overexpression of *OsCpn60α1* did not confer heat tolerance. Yet, it is essential for RbcL folding, as its failure can be lethal to seedlings^45^. The Cpn60β subunits were observed in two spots, while in drought, their content decreased in both spots, at a high temperature, the intensity of one spot did not change, and the other increased, while in double stress, the intensity of one spot increased, and the other decreased. Cpn60β plays a role in the folding of numerous proteins involved in the Calvin cycle and plastid division^46^. Moreover, in our experiment, RbcL was significantly upregulated only under high-temperature treatment, while combined stress led to its downregulation. Drought reduces Rubisco activity in various plants due to decreased protein levels or binding with sugar phosphate inhibitors^47–49^. This inhibition is reversible and mediated by Rubisco activase (RCA), which acts as a molecular chaperone to reverse it in an ATP-dependent manner^50^. However, RCA was downregulated across all treatments, especially under combined stresses, potentially hindering carboxylation. Heat stress minimally impacted potato yield, which may be attributed to significant Rubisco upregulation and slight RCA reduction. As was proven for some species, RCA may play a protective role for photosynthetic apparatus under heat^51–53^. These findings suggest that heat alone almost does not negatively affect Rubisco protective proteins; however, together with water deficit, it results in probable lowered protection of the crucial photosynthetic enzyme. Interestingly, none of these proteins were carbonylated, suggesting their resistance to this modification.

The carboxylation phase of the Calvin cycle is followed by the reduction and regeneration phases, both affected by drought and high temperatures. The reduction phase produces 3-phosphoglyceraldehyde, while the regeneration phase reconstructs carbon compounds^54^. In *Solanum tuberosum*, chloroplastic glyceraldehyde-3-phosphate dehydrogenase (GAPDH) exists as homotetramer GAPA₄ and heterotetramer A₂B₂, which include GAPA and GAPB subunits. Both forms convert 1,3-bisphosphoglycerate to 3-phosphoglyceraldehyde using NADPH. GAPB has a C-terminal domain with redox-regulatory functions, allowing modulation of enzyme activity based on the chloroplast redox state^55^. Our research showed varying levels of six GAPDH spots in response to abiotic stresses, suggesting different roles in carbohydrate metabolism. GAPA levels increased in all stresses at one spot while decreasing at another, possibly indicating post-translational modifications affecting its activity^56^. GAPB remained low, suggesting a lesser role under stress. As was shown by Simkin et al.^57^ Silencing GAPA reduced carbon assimilation by 73%, while silencing GAPB caused a 34% reduction, indicating that GAPA can compensate for GAPB loss. GAPDH also contributes to redox balance and carbon flow between pathways, enhancing plant resilience to oxidative stress^58^. Increased GAPDH levels under high temperatures suggest this stress was less severe for potato than the double stress, where we observed reduced GAPDH content and increased carbonylation.

Plastid aldolase (PA) and fructose-bisphosphate aldolase (FBA) 1, chloroplastic-like, are crucial for the Calvin cycle and carbohydrate metabolism by exhibiting FBA activity^59^. This activity regulates carbon flow, influencing whether products are used for sugar synthesis or RuBP regeneration^60^. Under drought and combined stresses, an increase in PA suggests efforts to restore photosynthetic balance. High carbonylation levels indicate oxidative damage; therefore, enhanced aldolase synthesis may be a compensation mechanism. In contrast, aldolase levels rise at high temperatures with low carbonylation, indicating improved oxidative stress resilience. Research on tobacco (*Nicotiana tabacum*) further highlights FBA’s role in boosting photosynthetic efficiency and growth^61^. However, our findings show a significant reduction of FBA 1 under double stress, suggesting decreased efficiency due to reduced biosynthesis and oxidative damage.

Chloroplastic transketolase (TK) also plays a critical role in regenerating RuBP and can influence the balance between the Calvin cycle and other metabolic pathways, especially under stress^62^. Our study identified differential regulation of TK, showing a significant increase in one isoform under high-temperature stress, while another decreased. Reduced TK content under drought and combined stress suggests a shift to conserve resources, with high carbonylation indicating compromised ability to regenerate CO₂ acceptors, thus limiting Calvin cycle efficiency. Studies by Henkes et al.^63^ demonstrated that tobacco plants with reduced plastid transketolase activity were more susceptible to stress. A reduction in TK activity led to decreased photosynthetic efficiency, affecting the production of ATP and NADPH.

Thus, while all stress treatments impaired the subsequent phases of photosynthesis, the extent of this inhibition varied significantly. High-temperature stress induced the mildest alterations, and several photosynthesis-related proteins even showed increased abundance, suggesting a partial maintenance of photosynthetic activity. In contrast, combined drought and heat stress triggered two concurrent effects: a marked downregulation of photosynthetic proteins and a pronounced increase in their carbonylation levels. This dual impact—reduced abundance and elevated oxidative modification—indicates a strong inhibition of photosynthetic processes under combined stress. Notably, such patterns were not observed under individual stresses, where protein carbonylation levels were generally lower, highlighting a unique metabolic response to the combined stress.Carbohydrate metabolism is essential for plant adaptation to abiotic stresses, as plants must quickly respond to maintain metabolic balance, energy production, and protect against oxidative damage^64–66^. Carbohydrates act as both an energy source and a substrate for protective compounds like reducing sugars and antioxidants^67^. Cytosolic fructose-1,6-bisphosphatase (FBPase) is crucial for gluconeogenesis, particularly under stress conditions such as drought and high temperatures. The shift towards catabolism, indicated by increased abbundance of carbonylated precursors and decreased mature FBPase levels, suggests limited storage sugar metabolism, impacting growth and photosynthetic efficiency^68,69^. Triosephosphate isomerase (TPI) plays a key role in glycolysis, facilitating the interconversion of dihydroxyacetone phosphate (DHAP) and 3-phosphoglyceraldehyde (G3P)^70^. TPI’s activity may be regulated under stress through modifications, but there are limited reports of its carbonylation. Increased carbonylation can decrease TPI activity, leading to DHAP accumulation and potentially toxic compound formation. Our studies indicated high carbonylation of TPI under drought conditions, which may lead to decreased sugar metabolism, energy production, and excess DHA accumulation.

Interestingly, changes in carbohydrate metabolism, in contrast to photosynthetic pathways, were most pronounced under single drought stress. These alterations seem to be regulated predominantly *via* post-translational modifications. Notably, significant carbonylation of the fructose-1,6-bisphosphatase (FBPase) precursor was observed, which coincided with a reduced abundance of its mature form. Additionally, carbonylation of triosephosphate isomerase (TPI) under drought stress was over 13-fold higher than in control conditions, highlighting a strong oxidative impact on this pathway. Interestingly, the presence of high temperature alongside drought appeared to mitigate this effect, suggesting that combined stress may paradoxically alleviate the inhibition of certain carbohydrate metabolic processes—possibly by altering the balance between oxidative damage and protein turnover.Potato plants possess sophisticated adaptive mechanisms at the cellular and molecular levels to mitigate stress effects^71^. Under high temperature and drought, they reduce primary metabolic protein biosynthesis while increasing stress response proteins like heat shock proteins (HSPs) and antioxidative enzymes. Notably, chloroplast HSPs protect the photosynthetic apparatus and maximize nutrient acquisition. Their levels were highest under combined stress and lowest with drought alone, highlighting the importance of HSPs in improving plant survival during such challenges. HSPs, typically produced in response to heat, aid in protein folding and protection against thermal damage. They also exist in non-stressed cells and play crucial roles throughout the cell cycle and development^72^. HSPs assist in the degradation of misfolded proteins and are categorized into families: HSP20 (also known as small HSP; sHSP), HSP60, HSP70, HSP90, and HSP100^73^.

Our study showed that the abundance of the chloroplastic-like HSP70 increased significantly under combined stress but underwent carbonylation, particularly under drought. This indicates HSP70’s vital role in dual stresses, facilitating protein folding and maintaining chloroplast function. Elevated levels of this protein enhance thermal tolerance and osmotic stress response in plants, as evidenced by studies on *Arabidopsis thaliana*, in which *cpHSC70-1* knockout mutants showed increased sensitivity to osmotic stress, and lowered activity of antioxidant enzymes which resulted in increased ROS accumulation. However, overexpression of this protein improved ROS scavenging capacity and increased the expression of stress-responsive genes^74,75^.

We also observed that chloroplastic HSP90-5 and sHSPs were also significantly influenced by stress occurrences. Although all sHSPs exhibited increased abundance in response to various stresses, the rise was least pronounced during drought, whereas they peaked during double stress. Similarly, chloroplastic HSP90-5 exhibited a notable increase under drought conditions, but some of the spots increased drastically under heat or double stress. A defining characteristic of sHSPs is their ability to bind substrate proteins without ATP, maintaining the stability of denatured proteins and preventing the formation of toxic aggregates^76,77^. Overexpression of *AtHSP21* in Arabidopsis enhanced heat resistance and improved the plant’s tolerance when re-exposed to heat stress^78^. Moreover, HSP90-5 is crucial for chloroplast development and embryogenesis. Its expression correlates with photosynthetic supercomplex accumulation and expression of genes involved in photosynthesis^79^. Functional studies indicate that mutations in the *Hsp90-5* lead to lethal effects, underscoring its essential role. Furthermore, this chaperone interacts with proteins essential for thylakoid membrane formation and plays a part in protein translocation into organelles. Hsp90-5 has been identified to interact with nuclear-encoded preprotein import intermediates during posttranslational import into isolated chloroplasts, such as stromal chaperones, Hsp93 and Hsp70^79,80^. Additionally, HSP83 abundance increased under all stresses, with the highest levels observed in response to heat. Its role in stress response is less understood, although it has shown increased accumulation in other species during stress conditions^81^.

In summary, it appears that the role of HSPs in drought was significantly limited. However, at high temperature and double stress, these were the proteins whose abundance increased the most, reaching values even over 23–24 times greater than in the control. Moreover, except for HSP70, they did not show increased carbonylation. The high HSP content at high temperature may be one of the reasons for the low total protein carbonylation and the slight decrease in carbonylation during double stress (compared to drought alone).

Changes in chaperones, particularly chaperonins, were observed under stress conditions. The chloroplast chaperone ClpB3 and a 20 kDa chaperonin showed variable levels. ClpB3 decreased during drought but increased under double stress. Its levels remained stable under high-temperature stress, despite its link to heat responses^82^. ClpB3 is prone to carbonylation, which is intensified during drought and double stress. ClpB3, part of the ClpB/Hsp101 family, aids in protein aggregate dissociation with stromal TRIGGER FACTOR 1 and sHSP 22E/F^83,84^. In tomato plants, inhibiting ClpB3 did not affect phenotype but reduced thermotolerance^85^. In Arabidopsis *clpb3*-knockout mutants, phenotypic changes included pale green coloration and lower PSII activity, which was lethal to seedlings^86–88^. Thus, ClpB3 primarily acts as a housekeeping chaperone in Arabidopsis and shifts localization during stress, suggesting a role in managing protein aggregates near thylakoid membranes^84^.

The ClpA subunit of ATP-dependent Clp protease was upregulated in all stress conditions. Its carbonylation increased in drought and double stress but decreased during heat, indicating a protective mechanism. Hsp70 and ClpB3, both upregulated under stress, cooperate with Clp protease to maintain protein quality^89^. Under oxidative stress, misfolded proteins aggregate, and while there is little direct evidence of carbonylated proteins being degraded by Clp, plastid Hsp70 and Clp systems it promotes plant survival migitating oxidative damage^90,91^.

Chaperonins appear to be particularly activated under dual stress, distinguishing the response to the coexistence of high temperature and drought from the same stresses occurring separately. The response to drought alone appears to be particularly different, with reduced levels of these proteins and their significant carbonylation. It should be emphasized that, unlike HSPs, chaperonins appear to be susceptible to oxidative damage.

The 20 kDa chaperonin functions as a co-chaperone of the 60 kDa chaperonin in chloroplasts. In potato plants, this chaperonin was identified in two spots. It was upregulated under double stress, but carbonylated in both double and drought stress. It may also act independently as a negative regulator in abscisic acid (ABA) signaling and FeSOD activation^92,93^. The 20 kDa chaperonin may participate in Ca^2+^-related signaling by binding calmodulin^94^. Changes in the abundance of the 20 kDa chaperonin, along with annexin and calreticulin-3-like proteins, suggest that Ca²⁺-dependent signaling is involved in the potato’s response to drought and heat stresses. Increased cytosolic Ca²⁺ concentration may link annexins to the plasma membrane, potentially regulating growth under mechanical stress and priming for further stress responses^95^. Overexpressing annexins may also increase ABA levels in tomatoes^96^. Plant calreticulins are crucial for binding Ca^2+^ and play a significant role in calcium storage in the endoplasmic reticulum. They are involved in modulating intracellular Ca^2+^ homeostasis and in the quality control of N-glycosylated proteins. In plants, there are three isoforms of calreticulins. Calreticulin-3 (CRT-3) provides innate immunity by conferring resistance to fungal and bacterial pathogens and is involved in the interaction between phytohormone signaling and Ca^2+^-mediated pathways, especially during stress response^97^. Our studies show that calreticulin-3-like protein was significantly down-regulated in both drought and combined stresses, likely leading to increased Ca^2+^ levels. Elevated cytosolic Ca^2+^ regulates ion channels, particularly by downregulating inward-conducting potassium channels while activating S-type anion channels, which mediate stomatal closure and inhibit their opening^98^. Thus, reduced calreticulin-3-like content under drought and dual stress may promote stomatal closure. At the same time, annexin is up-regulated, therefore its activation by Ca^2+^ is substantial, enabling plasma membrane protection against stress. In contrast, during heat stress, calreticulin is up-regulated while annexin level slightly decreased, indicating an alternative stress response to high temperatures, which stands in contrast to drought and double stressNotably, decreased carbonylation of NCED6 - a critical enzyme in ABA biosynthesis - was observed in all stresses, potentially leading to increased levels of this stress-related hormone, akin to observations in tomato^96^. However, it is worth mentioning that in drought conditions carbonylation is the lowest.

Environmental stresses of drought, high temperature, and their combination affect translation in potato chloroplasts. A decrease in the content of chloroplast elongation factors (EF) G and TuB was observed. This reduction may impact the protein elongation cycle, which is essential for tRNA translocation, involving the GTPase activity of EF-Tu and EF-G. The binding and hydrolysis of GTP in the presence of these factors facilitate the incorporation of aa-tRNA into the ribosome^99^. We also demonstrated that in response to stresses, chloroplastic ribosome binding factor PSRP1 is up-regulated. PSRP1 stabilizes ribosomal components but reduces tRNA binding, thus lowering translation capacity. It is recycled from the ribosome by the coordinated action of ribosome recycling factor and EF-G, suggesting that increased PSRP1 with decreased EF-G exacerbates translation inhibition under stress^100^. This mechanism seems to be, however, common for all studied stress combinations.

**In summary**, the study demonstrated that potato plants react differently to combined drought and high temperature stresses compared to each stress individually. Under combined stress, there was a significant decrease in key photosynthetic proteins, such as FNR, OEE1, and ATP synthase CF1, along with increased carbonylation, indicating greater oxidative damage. While ATP synthesis might be enhanced, double stress probably led to reduced photochemical efficiency and increased ROS synthesis, indicating inhibited photosynthesis. In contrast, high temperatures alone did not significantly affect Rubisco-protecting proteins, which remained stable with low carbonylation. Carbohydrate metabolism faced severe disruptions under drought alone, marked by highly increased carbonylation of FBPase precursors and TPI. However, high temperatures appeared to mitigate these effects when combined with drought. Importantly, under combined stress and high temperatures, there was a dramatic increase in chloroplastic HSPs levels (up to 24 times higher than control) with minimal carbonylation, suggesting lower oxidative damage. Chloroplastic chaperonins were also induced under combined stress but showed high susceptibility to carbonylation, indicating their vulnerability to concurrent stress factors. It seems that the response associated with maintaining chloroplast function is key to mitigating the effects of environmental stresses. Therefore, potato cultivars should be bred in such a way that breeding efforts can be directed towards achieving a strong response based on protecting chloroplasts from oxidative damage.

## Materials and methods

### Plant material

The study was carried out in the Potato Agronomy Department at the Plant Breeding and Acclimatization Institute-National Research Institute. The experiments were carried out on Polish potato (*Solanum tuberosum* L.) Lawenda cultivar. Considering the agronomic resistance to soil drought, this variety is considered relatively sensitive to soil drought^101^.

In the experiment, plants were grown in 14 l pots in control conditions in growth chamber. Pots were filled with a thin layer of gravel in the bottom and 12 l of universal vegetable soil substrate “Hollas” produced from peat with the addition of chalk at a pH range of 5.5–6.5 enriched with multicomponent fertilizer with formulation NPK 14-16-18 (*N* = 98, *P* = 49, K = 105 kg/ha) which means *N* = 2.45; *P* = 1.22; K = 2.61 g per plant). High-quality seed potatoes were used for the study. Tubers of 3–4.5 cm diameter were selected for planting. Two weeks before planting, seed potatoes were subjected to pre-sprouting and then ploughed into pot soil at 5–6 cm depth. A gum pipe was installed in each pot to improve soil aeration. Additionally, in phase 20 of the BBCH-scale of plant development, 10 g per plant of MIS-3 (Intermag) fertilizer (N 10.5, P_2_O_5_ − 8, K_2_O -16, MgO − 6, B − 1.8, Cu 8.7, Fe -7.0, Mn − 2.6, Mo-0.3, Zn − 0.6% m/m) was applied once. Plants were watered daily with an optimal tap water supply, that is over 70% of water field capacity measured by soil moisture tester (PAT.P. Nieuwkoop B.V. Aalsmer Holland). The vegetation chamber was equipped with six Hortilux Schreder Lamps with Philips light bulbs of 1600 W each. Air humidity was in the range 65–70%.

Two weeks after the initiation of the tuberization phase, the following combinations were used:

1. Control – optimal irrigation (70% of field water capacity) and temperature 20/16 °C.
2. Drought stress – without irrigation for 2 weeks during the tuberization period (40% of field water capacity) and optimal temperature 20/16 °C.
3. High-temperature stress (heat) - maintenance of elevated temperature (38/25°C) for 2 weeks during the tuberization period and optimal watering (70% of field water capacity).
4. Drought + high-temperature stress – without irrigation (40% of field water capacity) and maintenance of elevated temperature (38/25°C) for 2 weeks during tuberization period.

The stress conditions were selected based on the work of Mańkowska et al.^102^.

The fully expanded and mature leaves next to terminal leaflet leaves from third and fourth level from potato plants were sampled for biochemical analysis. Six leaves constituted one biological replicate. Three biological replicates were collected for biochemical analyses. The frozen tissues of potato leaves were ground to a fine powder with liquid nitrogen.

### Yield

After the end of the stress period, plants continued to be watered until the end of vegetation time (senescence). The yield of tubers of tested cultivar grown under optimal conditions (control combination) and stressed conditions (drought, heat and drought & heat) were given in grams per plant.

### RWC

The hydration of leaves was assessed as relative water content. First and second mature and fully expanded leaves next to terminal leaflet from the third level of compound leaf counting from the top of the plant, comparable in size, were sampled to assess relative water content (RWC). Ten leaves constituted one biological replicate. Measurements were conducted in three biological replicates and were performed between 9 and 11 a.m. Leaves were cut from the plant, weighed immediately (fresh weight, FW), floated in dark for 24 h to achieve turgidity (saturated weight, SW), then oven-dried (105 °C) for 24 h and weighed again (dry weight, DW). The RWC of leaves was calculated according to the formula: [(FW − DW)/(SW – DW)] × 100%.

### Leaf area

All compound leaves were cut from stems, and all leaves per plant were collected. Leaf area was measured with an LI-3100 A (LI-COR, Pullman, WA, USA) instrument. The result was given in cm^2^ per plant.

### Protein carbonylation

Protein carbonylation was determined by the method described by Levine et al.^103^. Samples of 200 mg of fresh leaves were homogenized at 4 °C in 3.0 ml of 50 mM sodium phosphate buffer pH 7.4, containing 1 mM EDTA. The crude extract was centrifuged at 6000×g for 10 min at 4 °C, and the supernatant was collected. The supernatant was then incubated with streptomycin sulphate (final concentration 1% w/v) on ice for 10 min. The extract was once again centrifuged (6000×g, 10 min, 4 °C), and the supernatant was collected. To 200 µl of supernatant, 800 µl of 10 mM DNPH (2,4-dinitrophenyl hydrazine) in 2.5 M HCl was added. Blank samples were prepared with 2.5 M HCl instead of DNPH/HCl. Samples were incubated for 1 h at room temperature in the dark with constant shaking. 1 ml of 20% (w/v) TCA was added to samples, and they were incubated on ice for 5 min. Afterward, samples were centrifuged at 10,000×g for 10 min at 4 °C. The pellet containing derivatized proteins was washed three times by suspension in 1 ml of an ethanol/ethyl acetate mixture (1:1), vortexing, and centrifuging at 10,000×g for 10 min at 4 °C. Washed pellet was dissolved in 1 ml of 6 M guanidine hydrochloride in potassium phosphate buffer (20 mM, pH 2.3). The absorbance was measured at 375 nm. The carbonyl content was calculated using a molar absorption coefficient for aliphatic hydrazones of 22,000 M^−1^ cm^−1^ and expressed in nmol carbonyl groups mg^−1^ protein. Total protein content was measured by spectrophotometric method^104^.

### Proteasome 20 S activity

Extracts were prepared by homogenization of 200 mg of fresh-weight plant samples by pulverization in a mortar with liquid nitrogen. Crude extracts were incubated for 30 min in a thermoshacker at 4 °C with 1.5 ml of 50 mM HEPS/KOH buffer pH 7.5 containing 2 mM MgCl_2_, 150mM NaCl, 10% (v/v) glycerol, and 1% (v/v) Triton-100. Samples then were centrifuged at 16,000×g for 30 min at 4 °C. Supernatants were collected. Protein content was measured in supernatants by Bradford’s method (1976). A sample volume containing 5 µg of protein was used for fluorometric measurement of proteasome 20 S activity by Acivity Assay Kit (Sigma-Aldrich, catalog nr: MAK172-1KT) according to the manufacturer’s guide.

### Statistical analysis

Representative data for the experiment (three independent biological repetitions) were presented as the means ± SD. The results were subjected to a two-way analysis of variance (ANOVA). The significant differences between experimental groups were determined using Tukey’s honest significant difference test at *p* < 0.05. Moreover, the homogeneity of variances in two-way ANOVA data was confirmed using the Brown-Forsythe test. The relationships between observed traits were estimated using Pearson’s linear correlation coefficients at *p* < 0.05. Statistical analysis was performed using GraphPad Prism 10 (GraphPad Software Inc.).

### Preparation of total protein extracts

Protein extraction and purification were performed to obtain proteome profiles. Leaves samples were ground in a mortar with liquid nitrogen. A sample of 150 mg of grounded tissue was purified according to^105^. The proteins were precipitated by adding ice-cold 10% (w/v) TCA/acetone. The sample was mixed and stored at − 20 °C for 30 min. Next, it was centrifuged at 16,000 × g for 30 min at 4 °C. The supernatant was discarded, and the pellet was washed twice with ice-cold TCA/acetone. After centrifugation, the tube was filled with 80% methanol containing 0.1 M ammonium acetate, mixed, and then centrifuged for 15 min. The supernatant was removed, and the pellet was washed with 80% acetone, mixed again, and then centrifuged, discarding the supernatant. The remaining pellet was air-dried at room temperature to remove any residual acetone, then dissolved in 0.6 mL of phenol (pH 8.0) and 0.6 mL of SDS buffer (0.1 M Tris-HCl, pH 8.0, containing 30% (w/v) sucrose, 5% (v/v) β-mercaptoethanol, and 2% (w/v) SDS) and mixed. After incubation at room temperature for 5 min, the water and phenolic phases were separated by centrifugation at 16,000 × g for 15 min at 4 °C. The upper (phenolic) phase was collected into a new tube. The extracted proteins were precipitated from the mixture by incubating overnight with 1.5 mL of 0.1 M ammonium acetate in 80% methanol at − 20 °C. The sample was then centrifuged for 30 min as described previously, and the supernatant was discarded. The pellet containing the extracted proteins was thoroughly washed with pure methanol and 80% acetone. After air-drying, the pellet was dissolved in isoelectric focusing (IEF) buffer, which consisted of 7 M urea, 2 M thiourea, 4% (w/v) CHAPS, and 40 mM DTT. The protein concentration was then determined photometrically at 595 nm according to^106^.

### Two-dimensional gel electrophoresis

Purified proteins were separated using two-dimensional gel electrophoresis (2D-PAGE). For the preparation of protein gels, a sample containing 120 µg of proteins was diluted to a total volume of 125 µL with isoelectric focusing (IEF) buffer. The IEF buffer consisted of 7 M urea, 2 M thiourea, 4% (w/v) CHAPS, 40 mM DTT, 0.5% (v/v) pH 3-10NL ampholytes, and 0.002% (w/v) bromophenol blue. This mixture was then subjected to IEF on 7-cm pH 4–7NL Immobiline DryStrips (Bio-Rad) using a Bio-Rad PROTEAN IEF focusing chamber, following the manufacturer’s recommendations. After IEF, the strips were incubated for 20 min in an equilibration buffer containing 50 mM Tris-HCl (pH 8.8), 6 M urea, 30% (v/v) glycerol, 2% (w/v) SDS, and 0.002% (w/v) bromophenol blue, with the addition of 1% (w/v) DTT. They were then equilibrated for another 20 min in the same buffer, but with 2.5% (w/v) iodoacetamide instead of DTT. After equilibration, the strips were sealed on top of SDS-PAGE gels (a 4% concentrating gel and an 11% separating gel, dimensions: 8.6 cm × 6.8 cm × 0.1 cm) using 0.5% (w/v) agarose in 0.1 M Tris-HCl (pH 6.8) containing 0.001% (w/v) bromophenol blue. The SDS-PAGE was run in 50 mM Tris-HCl (pH 6.8) buffer at 20 mA per gel for approximately 1 h, or until the blue dye front reached the bottom of the gel. The obtained gels were stained with colloidal Coomassie.

### Protein derivatization and transfer

A sample of 20 µg of purified proteins was subjected to IEF as previously described. The carbonyl groups were then derivatized with 2,4-dinitrophenylhydrazine (DNPH), following the method described by^107^, with some modifications. After IEF, the strips were incubated with gentle shaking in 5 mL of 10 mM DNPH dissolved in 2.5 M HCl for 20 min. They were subsequently washed five times, with each wash lasting 5 min, using an equilibration buffer. Next, the strips were incubated in an equilibration buffer containing 1% (w/v) DTT for 20 min and then in an equilibration buffer containing 2.5% (w/v) iodoacetamide for another 20 min. Once the equilibration steps were completed, the strips were sealed on top of the gels with 0.5% (w/v) agarose, and SDS-PAGE was performed as previously described.

### Detection of carbonylated proteins

To detect carbonylated proteins labeled with DNP, the proteins separated by SDS-PAGE were then transferred electrophoretically to a PVDF membrane using a Bio-Rad transfer system, following the manufacturer’s protocol. The membrane was blocked by incubation at room temperature in 5% (w/v) non-fat milk solution in PBS buffer (pH 7.4, containing 137 mM sodium chloride, 10 mM phosphate, and 2.7 mM potassium chloride) with 0.5% (v/v) Tween 20 for 1 h. After 3 washes with PBST, a wash with PBS buffer without Tween 20 was performed, and the proteins were detected by incubating the membrane with rabbit anti-DNP antibodies (Sigma; 1:2,000 dilution) followed by incubation with alkaline phosphatase-conjugated goat antibodies against rabbit (Sigma; 1:30,000 dilution). To visualize the blots, a standard NBT/BCIP solution was used, prepared by mixing 10 mL of 0.1 M Tris-HCl buffer (pH 9.5), 0.1 M NaCl, 0.05 M MgCl_2_, along with 1.5 mg of BCIP and 3 mg of NBT.

### Proteome profiles analysis

Four biological replicates of each experimental variant were conducted, encompassing protein purification, two-dimensional electrophoresis (2DE), membrane transfer, and immunochemical staining (*n* = 4). Gels and PVDF membranes were scanned using an Image Scanner III (GE Healthcare), and their digital images were analyzed with Delta2D Version 2.0 software (DECODON GmbH, Greifswald, Germany). Differential proteins were identified by comparing plants subjected to drought, high temperature, and a combination of these stresses with control leaves within the cultivar. After correcting for positional spot variations, a virtual fused image was created to condense the information from all images into one composite image. This was followed by the detection of a consensus spot pattern. For spot quantitation, based on size and intensity, a standard procedure embedded in the software was applied to all membrane images from the experiment. Normalized spot intensities were derived by relating the intensity of each single spot to the total intensity of all detected spots in the gel or membrane image. The accuracy of gel or membrane identity between biological replicates was assessed using principal component analysis (PCA), which served as both a quality control step and a means to compare all experimental variants. The selection of differential proteins was based on mean spot intensity and was evaluated using one-way ANOVA with an adjusted Bonferroni correction (critical p-value < 0.05). Images of selected spots on membranes were overlaid onto the corresponding gel images, and the selected spots were then excised from the gels for identification purposes.

### Identification of selected proteins

The LC–MS/MS analysis was performed commercially by the Mass Spectrometry Laboratory of the Institute of Biochemistry and Biophysics at the Polish Academy of Sciences (IBB PAS) using an Orbitrap Exploris 480 mass spectrometer (Thermo Fisher Scientific). The gel spots were excised and incubated in 100 µL of a destaining solution, which consisted of 50% (v/v) 50 mM ammonium bicarbonate and 50% (v/v) acetonitrile (ACN). After drying, the gel pieces were incubated in 100 µL of pure ACN until they shrank. Next, the cysteine (Cys) residues were reduced by incubating the gel pieces in 50 µL of 10 mM dithiothreitol (DTT) dissolved in 100 mM ammonium bicarbonate. Following drying and shrinking, the Cys residues were alkylated by incubating the gel pieces in 50 µL of 50 mM iodoacetamide in 100 mM ammonium bicarbonate. The gel pieces were then washed twice with 100 mM ammonium bicarbonate and dried in pure ACN. After shrinking the gel pieces in pure ACN, trypsin digestion was performed overnight at 37 °C using a trypsin solution at a concentration of 10 ng/µL in 25 mM ammonium bicarbonate. Finally, the peptides were extracted from the gel pieces three times with a solution containing 0.1% trifluoroacetic acid (TFA) (v/v) and 2% (w/v) ACN.

The NCBI database for *Solanum tuberosum*, which contains 35,618 sequences, was utilized (NCBIprot 20191214; 229,636,095 sequences; 83,676,080,993 residues; accessed on November 4, 2020). The database was accessed via the MASCOT server, and the MS/MS Ion Search was performed. The peptide mass tolerance was set to ± 5 ppm, while the fragment mass tolerance was set to ± 0.01 Da, allowing for up to 2 missed cleavages. Carbamidomethyl was designated as a fixed modification, and oxidation was included as a variable modification.

### Protein interaction data analysis

The prediction of functional networks of proteins was performed using STRING 12.0 software (http://string-db.org), a database of known and predicted protein interactions (PPIs)^108^. PPIs were determined using a minimum required interaction score of 0.4.

## Supplementary Information

Below is the link to the electronic supplementary material.

Supplementary Material 1

## Data Availability

The datasets generated during and/or analysed during the current study are available from the corresponding author on reasonable request.
