# Supplementary material for: Changes in proteome and protein carbonylation in potato (Solanum tuberosum L.) under single and combined abiotic stresses
Source: Sci Rep. 2025 Oct 28;15:37645. doi: 10.1038/s41598-025-21439-y (PMC12569099; doi:10.1038/s41598-025-21439-y)
Supplement: Supplementary file 1 — Supplementary Material 1 [file 41598_2025_21439_MOESM1_ESM.zip › supplementary legends.docx]

Supplementary Information:

Supplement 1. Representative images of the obtained gels with marked significantly differentiating proteins. Each spot is assigned a unique number. The selection of differential proteins was based on mean spot intensity and was evaluated using one-way ANOVA with an adjusted Bonferroni correction (critical p-value < 0.05).

Supplement 2. Principal component analysis: a - for obtained gels; b - for obtained western blots imaging carbonylated proteins. In each combination, 4 obtained images were used. The color of the points corresponds to the tested combination: red - control; blue - drought; yellow - high temperature; green - double drought and high temperature stress.

Supplement 3. Representative images of the obtained western blots on PVDF membrane with marked significantly differentiating carbonylated proteins. Each spot is assigned a unique number. The selection of differential proteins was based on mean spot intensity and was evaluated using one-way ANOVA with an adjusted Bonferroni correction (critical p-value < 0.05).
