# Supplementary figures and images for: Changes in proteome and protein carbonylation in potato (Solanum tuberosum L.) under single and combined abiotic stresses
Source: Sci Rep. 2025 Oct 28;15:37645. doi: 10.1038/s41598-025-21439-y (PMC12569099; doi:10.1038/s41598-025-21439-y)

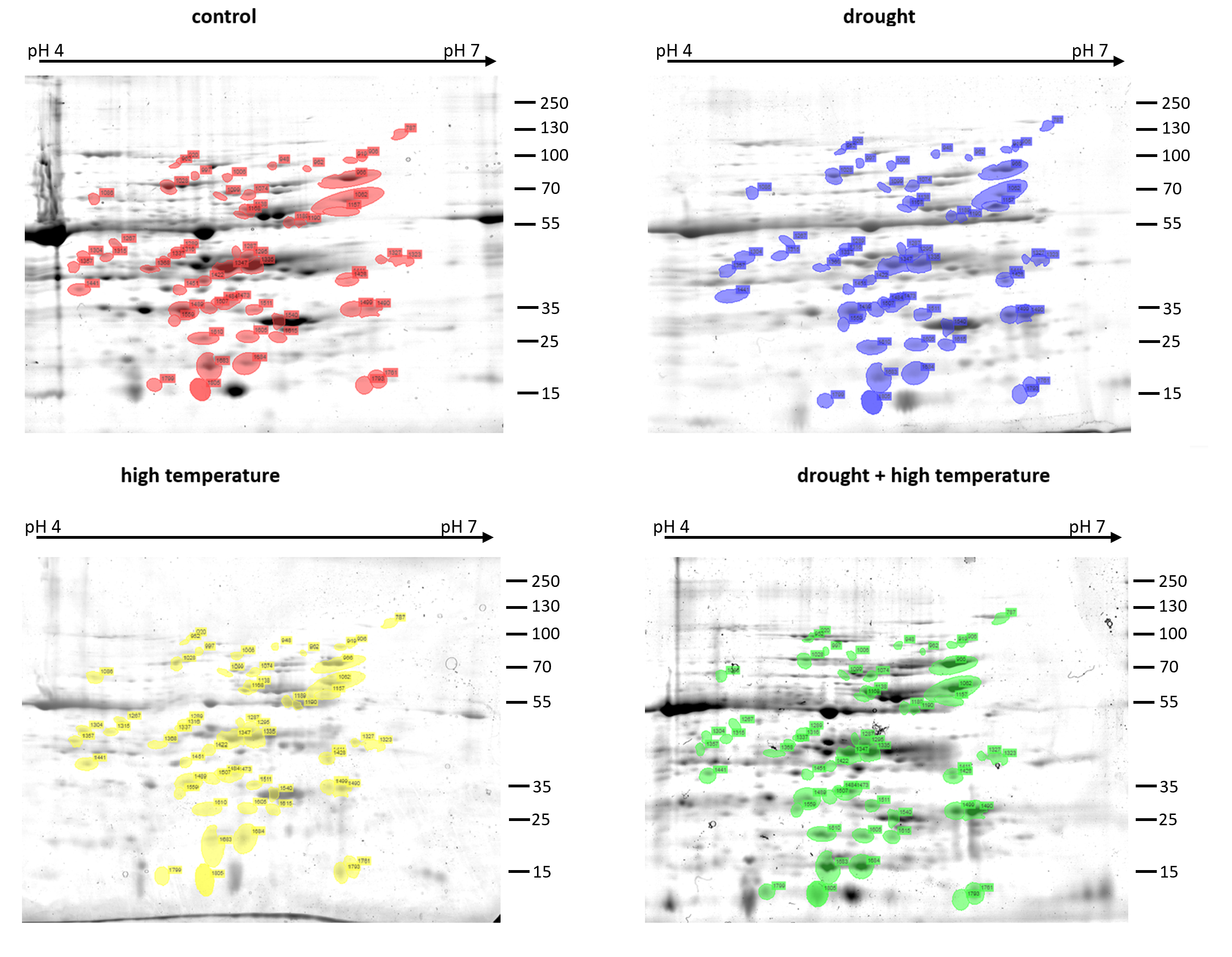

Supplement: Supplementary file 1 — Supplementary Material 1 [file 41598_2025_21439_MOESM1_ESM.zip › Supplement 1.tif]

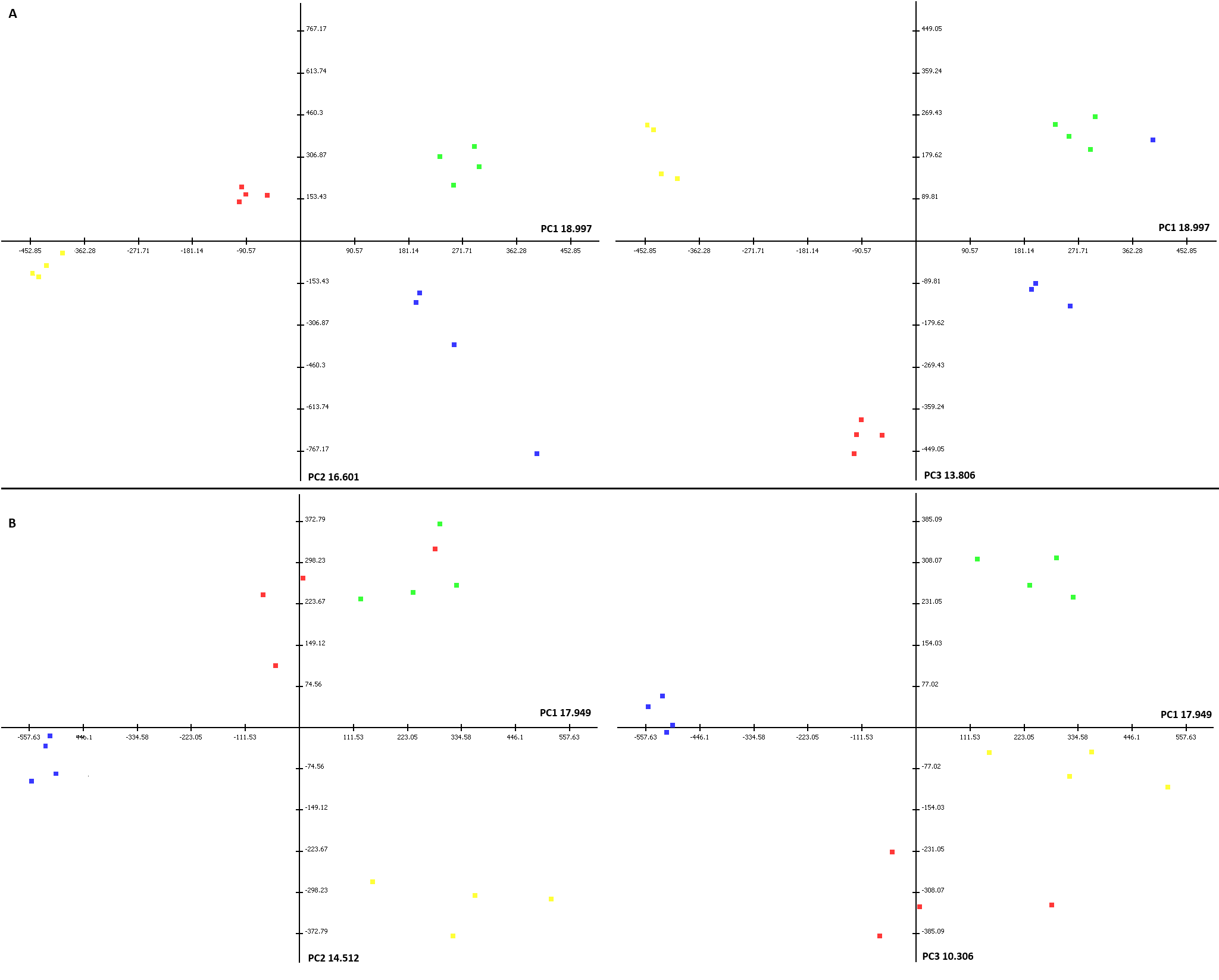

Supplement: Supplementary file 1 — Supplementary Material 1 [file 41598_2025_21439_MOESM1_ESM.zip › Supplement 2.tif]

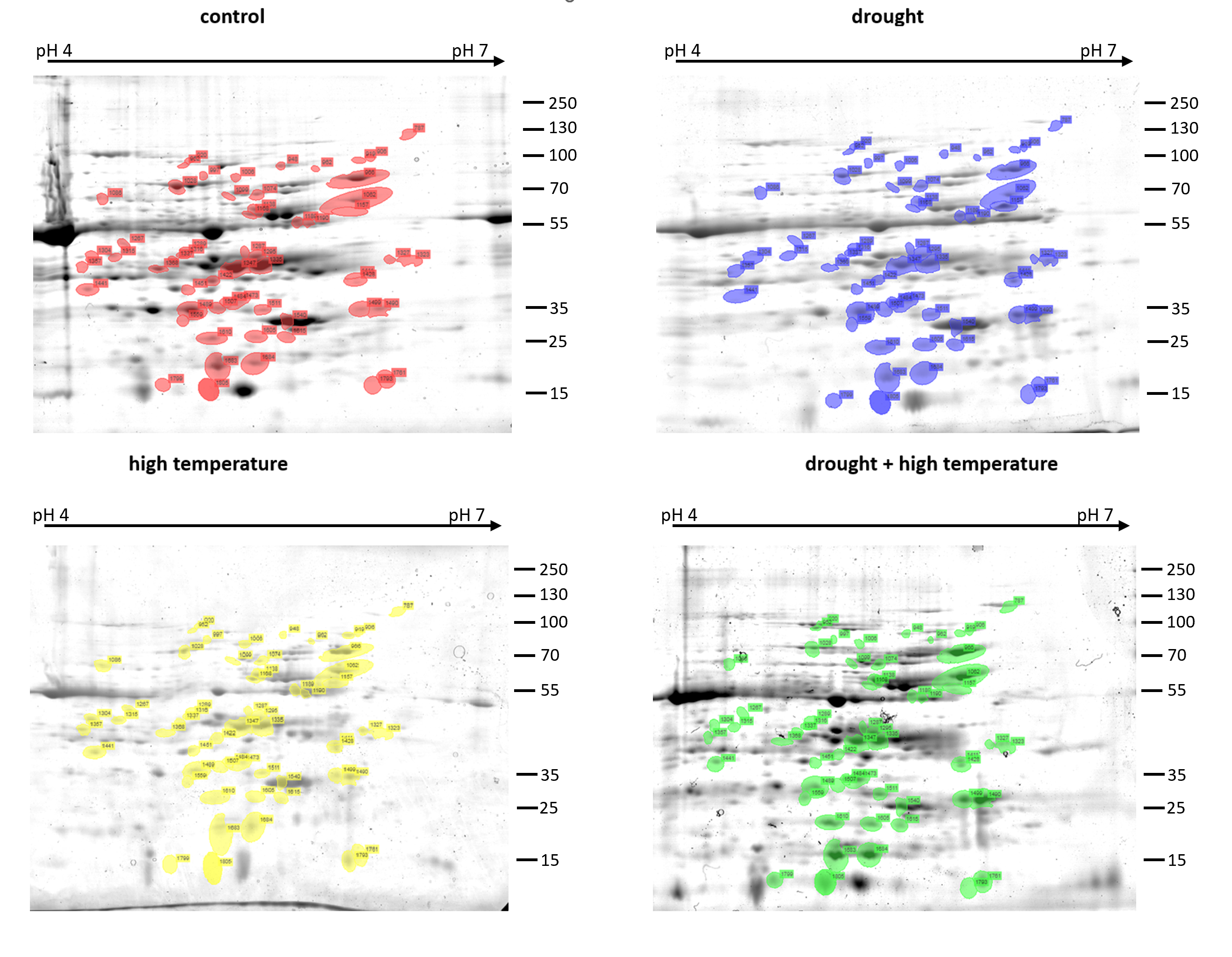

Supplement: Supplementary file 1 — Supplementary Material 1 [file 41598_2025_21439_MOESM1_ESM.zip › Supplement 3.tif]
